# Supplementary material for: Transgenic Maize of ZmMYB3R Shapes Microbiome on Adaxial and Abaxial Surface of Leaves to Promote Disease Resistance
Source: Microorganisms. 2025 Feb 7;13(2):362. doi: 10.3390/microorganisms13020362 (PMC11858687; doi:10.3390/microorganisms13020362)
Supplement: Supplementary file 1 [file microorganisms-13-00362-s001.zip › microorganisms-3424721-supplementary.pdf]

Supplementary table

Table S1 Diversity evaluation of the metagenome analysis from the phyllosphere of the adaxial and adaxial leaf surfaces of wild-type (WT) and transgenic (OE) maize.

Sample Sites	WT-adaxial	OE-adaxial	WT-abaxial	OE-abaxial
	Uploading Information			
bp Count	10824340373	10282641262	9887670864	10713144779
Sequence Count	71684373.33	68096962	65481264	70947978.67
Mean Sequence Length	151	151	151	151
Mean GC Percent (%)	52.4	52.0	52.9	55.4
	Post QC Information			
bp Count	10443297718	9868929327	9519769320	10265418650
Sequence Count	69482259.33	65701522	63314571.3	68287990
Mean Sequence Length	150	150	150	150
Mean GC Percent (%)	52.2	51.7	52.6	55.1

WT-adaxial, adaxial surfaces of leaves from wild-type maize; OE-adaxial, adaxial surfaces of leaves from transgenic maize (*ZmMYB3R*-overexpressing maize). WT-abaxial, abaxial surfaces of leaves from wild-type maize; OE-abaxial, abaxial surfaces of leaves from *ZmMYB3R*-overexpressing maize.

Table S2 Effects of niche and genotype on bacteria and fungi at the phylum level

Bacterial Phyla	Armatimonadetes	Deinococcus Thermus	Planctomycetota	Cyanobacteria	Acidobacteria	Chloroflexi	Firmicutes	Bacteroidota	Actinobacteria	Proteobacteria
WT_adaxial	69.24±5.55b	12.12±4a	10.69±1.86a	2.37±0.8a	0.43±0.15a	0.4±0.12a	0.83±0.43a	0.09±0.01a	0.38±0.33a	0.01±0b
OE_adaxial	79.71±4.9ab	9.22±2.87ab	2.13±0.89b	3.7±3.21a	0.25±0.1a	0.8±0.32a	0.21±0.08b	0.13±0.04a	0.12±0.08a	0.04±0.01a
WT_abaxial	71.24±5.31b	11.19±2.19ab	8.4±3.45a	4.98±1.82a	0.44±0.22a	0.46±0.14a	0.12±0.01b	0.08±0.03a	0.18±0.08a	0.01±0.01b
OE_abaxial	88.81±3.91a	4.39±1.98b	0.79±0.52b	1.83±0.69a	0.1±0.04a	0.79±0.29a	0.06±0.01b	0.09±0.03a	0.04±0.05a	0.02±0.01ab
Niche	**0.0250**	**0.0250**	0.2002	0.1495	**0.0104**	**0.0163**	0.2623	**0.0039**	**0.0374**	**0.0039**
Transgene	0.4433	0.4433	0.4433	0.4433	0.4433	0.4433	0.4433	0.4433	0.4433	0.4433
Niche*Transgene	0.7488	0.1495	0.3367	**0.0039**	0.8728	0.3367	0.6310	0.3367	0.2002	0.5218

Fungal Phylum	Blastocladiomycota	Oomycota	Mucoromycota	Ascomycota	Basidiomycota
WT_adaxial	13.1±7a	0.64±0.26a	0.07±0.06a	0.05±0.05a	0.02±0.03a
OE_adaxial	1.46±0.13b	0.4±0.13a	0.03±0.04a	0.05±0a	0.01±0.01a
WT_abaxial	5.14±1.99ab	0.55±0.23a	0.08±0.07a	0.02±0.01a	0±0a
OE_abaxial	1.87±1.06b	0.52±0.34a	0.1±0.13a	0.05±0.01a	0±0a
Niche	0.3333	0.2623	**0.0039**	**0.0374**	**0.0163**
Transgene	0.1667	0.1972	0.4533	0.3019	0.3019
Niche*Transgene	0.5189	1.0000	0.5218	0.1093	0.1093

WT-adaxial, adaxial surfaces of leaves from wild-type maize; OE-adaxial, adaxial surfaces of leaves from transgenic maize (*ZmMYB3R*-overexpressing maize). WT-abaxial, abaxial surfaces of leaves from wild-type maize; OE-abaxial, abaxial surfaces of leaves from *ZmMYB3R*-overexpressing maize. Bold means $p<0.05$.

Table S3 Effects of niche and genotype on bacteria and fungi at the species level

WT_adaxial	0.99±0.21a	1.07±0.31a	0.84±0.62a	0.65±0.5a	0.52±0.5a	0.61±0.19a	0.07±0.03a	0.78±0.27a	0.05±0.03a	0.17±0.16a
OE_adaxial	0.81±0.07ab	2.24±1.14a	0.82±0.3a	0.32±0.28a	1.12±0.6a	1.24±0.51a	0.11±0.01a	1.55±0.71a	0.06±0a	0.31±0.1a
WT_abaxial	0.78±0.35ab	1.34±0.35a	0.82±1.11a	0.54±0.65a	0.9±0.67a	0.71±0.17a	0.08±0.04a	0.96±0.24a	0.04±0.01a	0.17±0.07a
OE_abaxial	0.32±0.24b	2.45±1.24a	2.8±2.9a	1.12±1.46a	0.56±0.33a	1.43±0.32a	0.09±0.02a	1.51±0.93a	0.08±0.04a	0.29±0.04a
Niche	0.0547	**0.0374**	0.1093	0.1495	**0.0039**	0.4233	0.8728	0.2623	0.0547	0.1495
Transgene	0.4433	0.4433	0.4433	0.4433	0.4433	0.4433	0.4433	0.4433	0.4433	0.4433
Niche*Transgene	1.0000	1.0000	0.7488	0.4233	0.7488	0.6310	0.8728	0.6310	0.6310	0.0374
Fungal species	Venturia nashicola	Bipolaris sorokiniana	Ustilago trichophora	Rhizopus arrhizus	Hortaea werneckii	Moesziomyces aphidis	Moesziomyces antarcticus	Penicillium oxalicum	Bipolaris maydis	Tilletiaria anomala
WT_adaxial	23±3.55b	6.35±1.19a	1.28±0.39a	1.13±0.2a	0.91±0.2a	3.13±1.08a	6.36±3.27a	0.45±0.11a	0.3±0.1a	0.83±0.26a
OE_adaxial	36.41±13.73ab	1.82±0.89b	1.12±1.01a	2.41±0.6a	2.04±0.33a	2.29±2.06a	0.45±0.12b	2.06±1.89a	0.08±0.05b	0.46±0.38a
WT_abaxial	37.46±5.44ab	4±1.52ab	1.5±0.41a	1.4±0.53a	1.38±0.64a	2.8±0.6a	2.18±1.17ab	0.42±0.25a	0.3±0.03a	0.61±0.08a
OE_abaxial	55.72±13.98a	1.33±0.71b	0.89±0.75a	1.32±1.16a	1.17±0.91a	1.39±1.17a	0.48±0.24b	0.73±0.5a	0.07±0.09b	0.32±0.19a
Niche	0.0547	**0.0039**	0.0547	**0.0039**	0.1093	0.2623	0.3367	0.3367	**0.0065**	0.0782
Transgene	0.4433	0.4433	0.4433	0.4433	0.4433	0.4433	0.4433	0.4433	0.4433	0.4433
Niche*Transgene	0.3367	0.6310	0.5218	0.5218	0.3367	0.6310	0.4233	0.8728	0.2623	0.0250

WT-adaxial, adaxial surfaces of leaves from wild-type maize; OE-adaxial, adaxial surfaces of leaves from transgenic maize (*ZmMYB3R*-overexpressing maize). WT-abaxial, abaxial surfaces of leaves from wild-type maize; OE-abaxial, abaxial surfaces of leaves from *ZmMYB3R*-overexpressing maize. Bold means $p<0.05$.

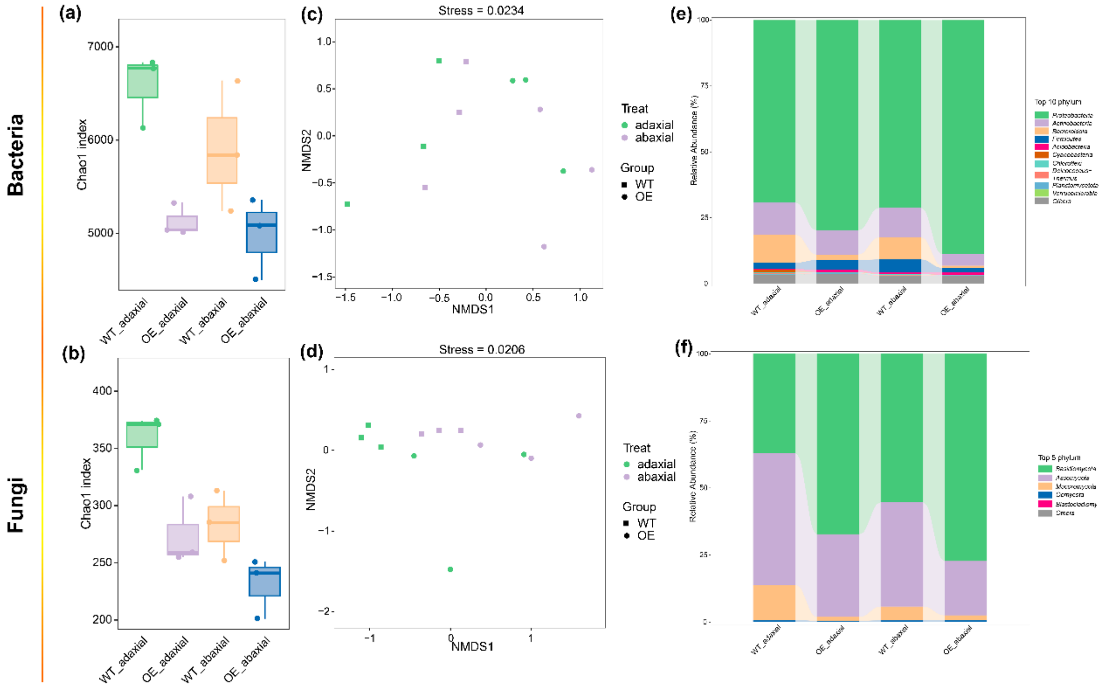

Figure S1 Bacterial and fungal communities in the maize phyllosphere. Chao1 index of (a) bacterial and (b) fungal communities on the adaxial and abaxial surfaces of maize leaves (one-way analysis of variance (ANOVA), n = 3, P < 0.05). Non-metric multi-dimensional scaling (NMDS) ordinations based on the Bray-Curtis similarity revealed differences in the structure of (c) bacterial and (d) fungal communities on the adaxial and abaxial surfaces of WT and *ZmMYB3R*-OE leaves (permutational ANOVA (PERMANOVA), n = 3, P < 0.05). Relative abundances of (e) bacterial and (f) fungal phyla. OE represents transgenic plants with overexpression of the *ZmMYB3R* gene, and WT represents control varieties.

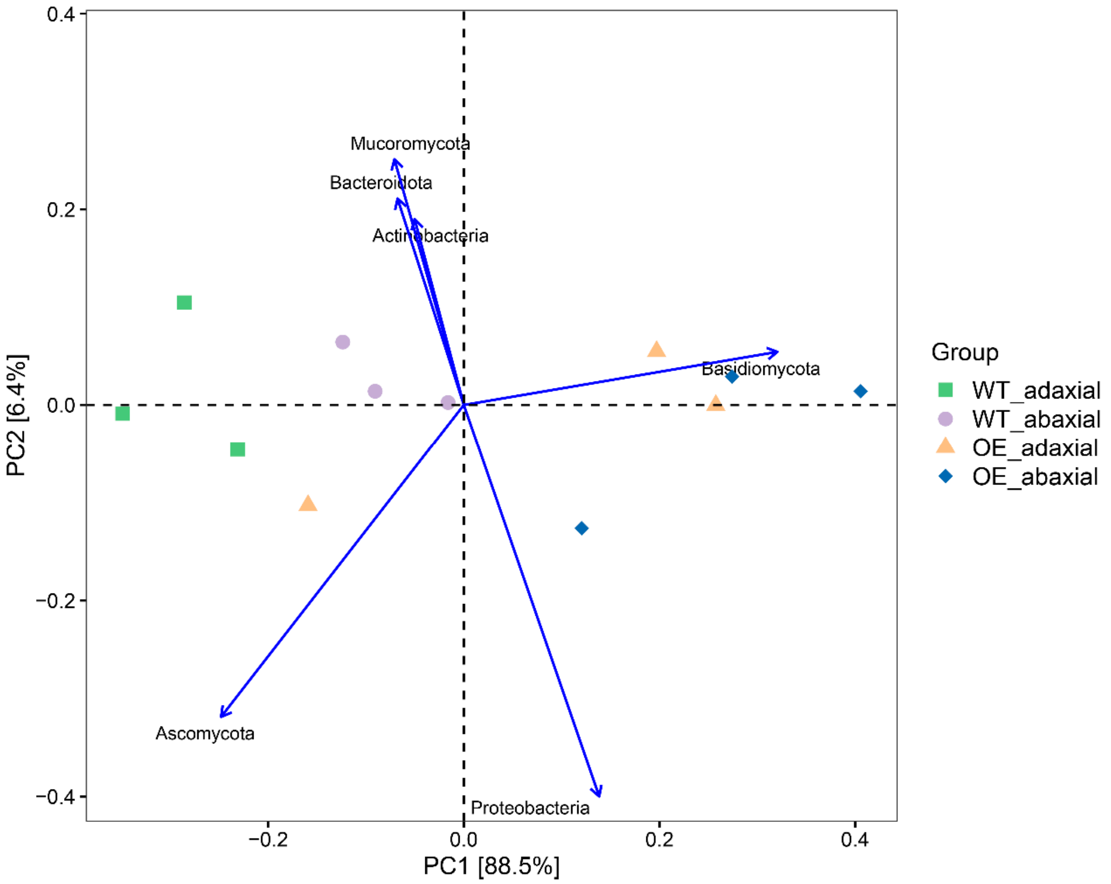

Figure S2 Principal component analysis (PCA) graph of average phyllosphere microbial community phyla. WT-adaxial, adaxial surfaces of leaves from wild-type maize; OE-adaxial, adaxial surfaces of leaves from *ZmMYB3R*-overexpressing maize. WT-abaxial, abaxial surfaces of leaves from wild-type maize; OE-abaxial, abaxial surfaces of leaves from *ZmMYB3R*-overexpressing maize.

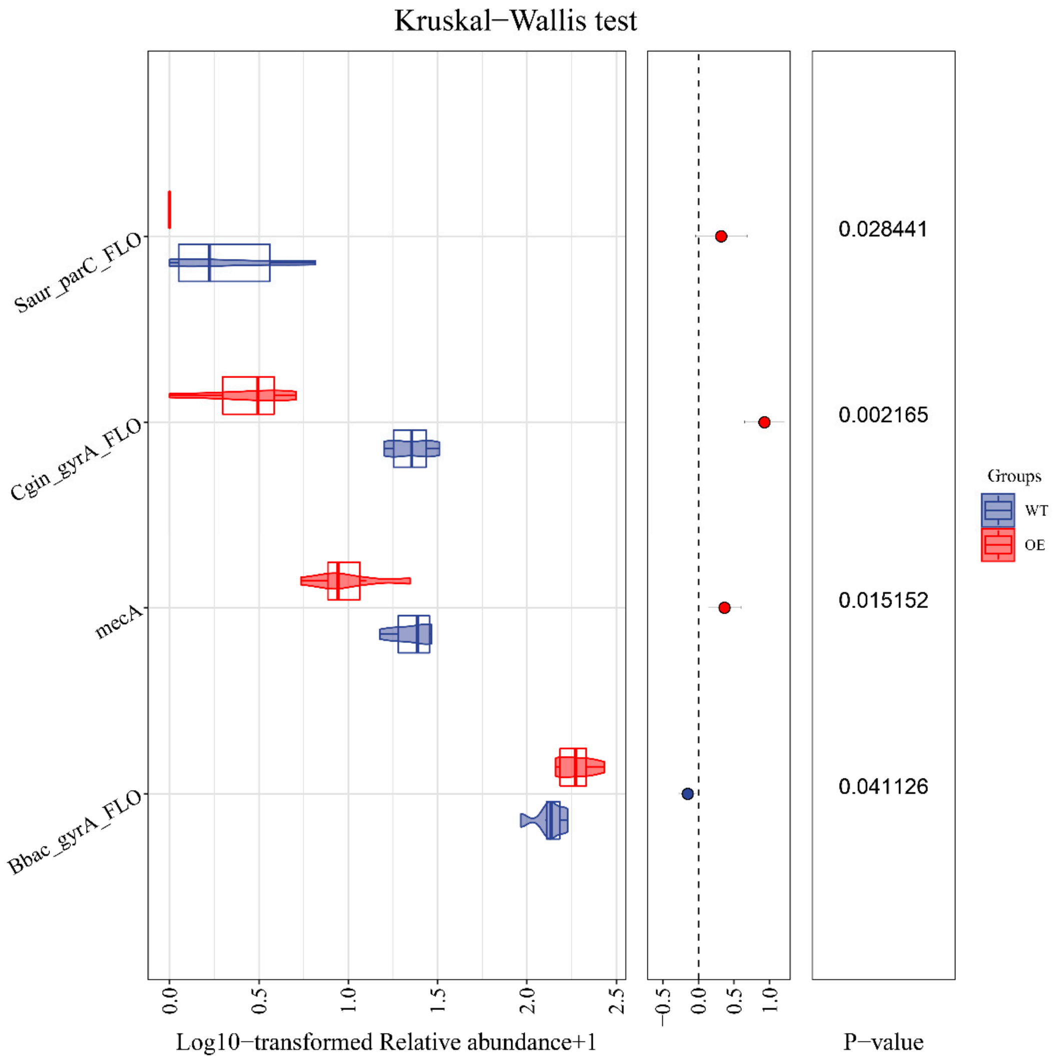

Figure S3 Relative abundances of resistance genes that significantly (Anova, p < 0.05) differed in the phyllosphere of wild-type (WT) and transgenic (OE) maize.

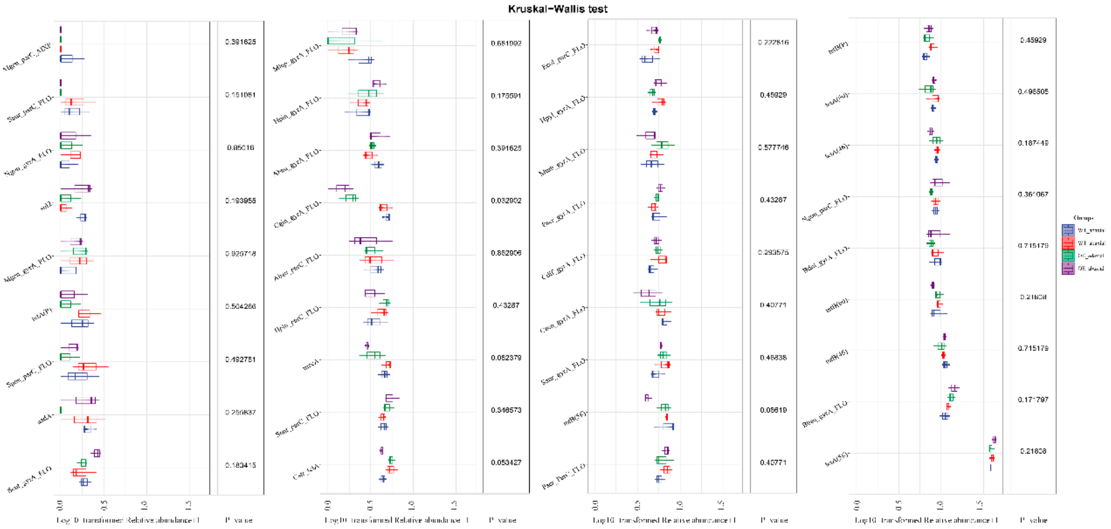

Figure S4 Relative abundances of resistance genes in the phyllosphere of each surface of wild-type (WT) and transgenic (OE) maize. WT-adaxial, adaxial

surfaces of leaves from wild-type maize; OE-adaxial, adaxial surfaces of leaves from *ZmMYB3R*-overexpressing maize. WT-abaxial, abaxial surfaces of leaves from wild-type maize; OE-abaxial, abaxial surfaces of leaves from *ZmMYB3R*-overexpressing maize.

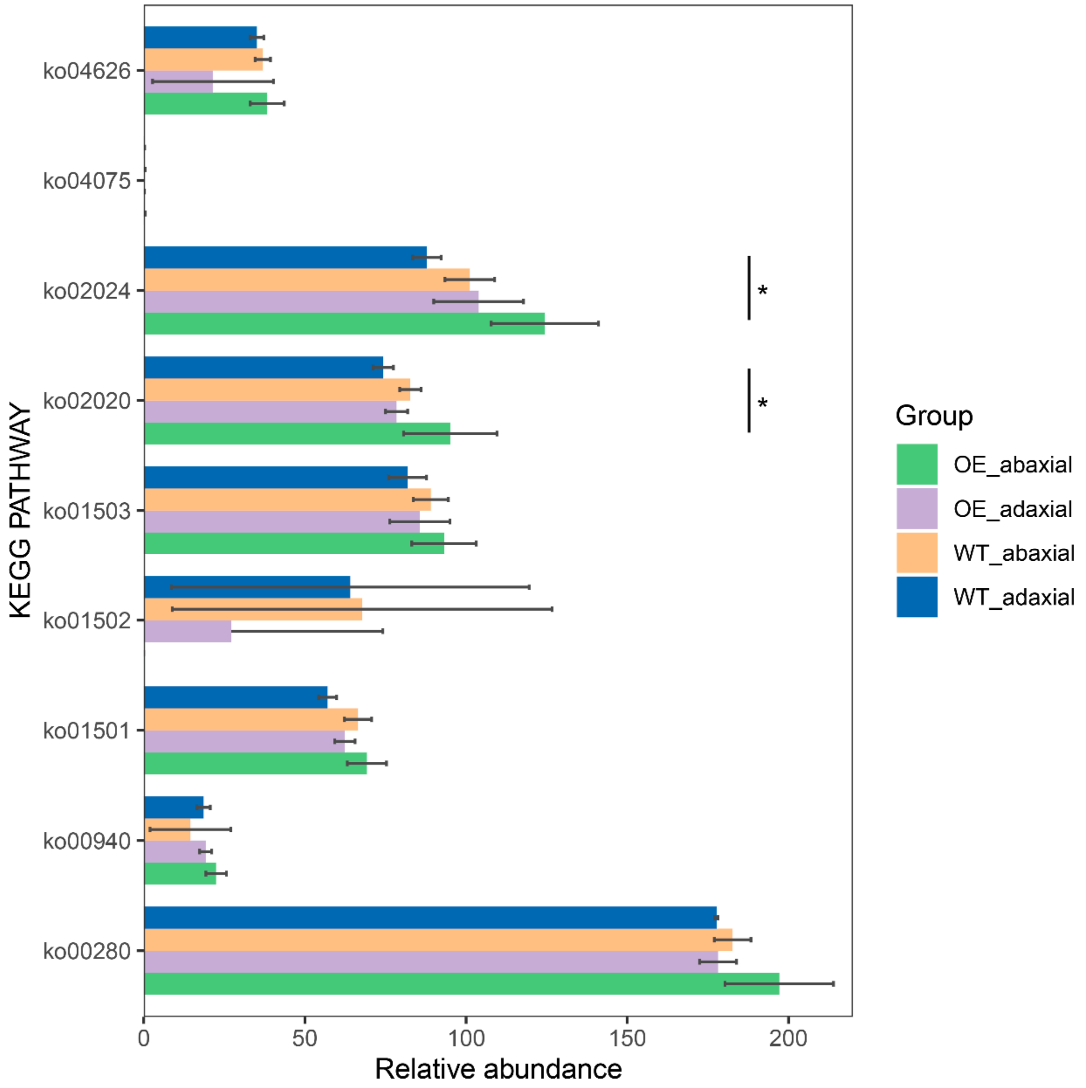

Figure S5 Disease-resistance metabolic pathways enriched by functional genes. WT-adaxial, adaxial surfaces of leaves from wild-type maize; OE-adaxial, adaxial surfaces of leaves from *ZmMYB3R*-overexpressing maize. WT-abaxial, abaxial surfaces of leaves from wild-type maize; OE-abaxial, abaxial surfaces of leaves from *ZmMYB3R*-overexpressing maize.
